# The Effect of Muscle Graft With Nerve Growth Factor and Laminin on Sciatic Nerve Repair in Rats

**DOI:** 10.32598/bcn.9.10.145

**Published:** 2019-07-01

**Authors:** Mehrzad Jafari, Hamdollah Delaviz, Somayeh Torabi, Jamshid Mohammadi, Izadpanah Gheitasi

**Affiliations:** 1. Cellular and Molecular Research Center, Yasuj University of Medical Sciences, Yasuj, Iran.; 2. Herbal Medicine Research Center, Yasuj University of Medical Sciences, Yasuj, Iran.

**Keywords:** Peripheral nerve, Muscle graft, Growth factor, Laminin, Rats

## Abstract

**Introduction:**

Peripheral nerve injury is one of the most common damages that lead to physical disability. Considering the similarity between the coatings of skeletal muscles and nerve fibers, we conducted this research to determine the effect of muscle graft with Nerve Growth Factor (NGF) and Laminin (L) on nerve repair.

**Methods:**

We cut a 10-mm length of the sciatic nerve from 42 female Wistar rats (Weight: 200±250 g) and equally divided the rats into three groups. In the muscle graft+NGF+laminin group, the degenerated skeletal muscle was sutured with proximal and distal ends of the transected sciatic nerve. Then, NGF (100 ng) and laminin (1.28 mg/mL) were injected into the muscle graft. In the muscle graft group, normal saline was injected into the muscle graft. In the control group, 10 mm of the sciatic nerve was removed without any treatment. Functional recovery was assessed based on Sciatic Functional Index (SFI). Also, tracing motor neurons and histological studies were performed to evaluate nerve repair. The obtained data were analyzed by ANOVA test.

**Results:**

The Mean±SD SFI value significantly increased in the muscle graft+NGF+laminin (−76.6±2.9) and muscle graft (−82.1±3.5) groups 60 days after the injury compared to the control group. The Mean±SD number of labeled motor neurons significantly increased in the muscle graft+NGF+laminin (78.6±3.1) and muscle graft (61.3±6.1) groups compared to the control group (P<0.001). The mean number of myelinated axons in the distal segments of the muscle graft+NGF+laminin increased significantly compared to the muscle graft group.

**Conclusion:**

These findings suggest that muscle graft followed by NGF and laminin administration have therapeutic effects on nerve repair.

## Highlights

- Functional or sensory loss may follow peripheral nerve injury.
- Surgical techniques and stimulating nerve growth are necessary to protect nerve regeneration.
- The oriented basement membrane and extracellular matrix component of the autologous degenerated skeletal muscle could conduit the regenerating nerve fibers.
- Nerve growth factor (NGF) has a vital role in the nerve growth and survival of neurons and increase nerve regeneration and remyelination after nerve defect.
- Combination of autologous degenerated skeletal muscle with NGF and laminin has promising treatment in the peripheral nerve injury

## Plain Language Summary

Peripheral nerve injury results in disability, limitation of daily living, and higher expenses for the family. This lesion requires a comprehensive treatment that provides both a conduit for nerve conduction and essential materials for nerve growth. Epimysium around the muscle fiber and epineurium around the nerve fiber have the same structure. Both of them are necessary for nerve and muscle survival and their function. In this study, the muscle fiber was removed from the autologous degenerated skeletal muscle and basement membrane, and epimysium was used as a conduit for the interrupted nerve. NGF is a neuropeptide that participated in the regulation of nerve growth, adhesion, migration, neuronal proliferation, and regeneration and remyelination of nerve fiber. Laminin is a component of the basement membrane and act as a surface substrate for nerve repair and produced mainly by Schwann cells. Histological of the distal segment after the transplantation in the region demonstrated that axons could grow and reach the distal part. The study results indicate autologous degenerated skeletal muscle along with NGF and Laminin could provide a useful environment for axonal regeneration and functional recovery.

## 1. Introduction

Peripheral nerve injury is one of the most common damages; many lead to permanent disabilities and neuropathic pain (Sullivan, Dailey, Duncan, Abel, & Borlongan, 2016). The most severe of which is called neurotmesis that results in the loss of nerve trunk continuity, myelin sheath, and the surrounding connective tissues. If there is no gap between the cut endings of the nerve, or if the gap is short, the two nerve endings can be directly sutured, and the result of healing will depend on whether the nerve bundles at the cut endings to adhere to each other correctly (Mafi, Hindocha, Dhital, & Saleh, 2012).

Although some drugs such as cyclosporine A and melatonin have useful effect for axonal regeneration and sprouting effect for peripheral nerve injury (Roozbehi, Joghataie, Mehdizadeh, Mirzaei, & Delaviz, 2012; Turgut & Kaplan, 2011), in the long gap of peripheral nerve injuries, diversion of axonal buds at the injury site is a significant problem that can influence nerve repair (Panagopoulos, Megaloikonomos, & Mavrogenis, 2017). Therefore, this gap must be bridged so that the generated axons can be guided toward the distal stump and target organ. In these cases, the material used for bridging the gap can influence the result of the repairing process (Panagopoulos et al., 2017).

Polyvinylidene fluoride channel as a conduit for bridging the peripheral nerves defects with nerve growth factor (NGF) and collagen gel could provide a favorable matrix for axonal regrowth (Delaviz et al., 2011). The basal membrane could stimulate and create guidance channels for regrowth of axons toward the target tissue (Kang, Hu, Wang, & Wang, 2015). It has been shown that muscle basal lamina grafts can be a guidance route for the injured axons toward the distal segment of the endoneurial tubes (La Fleur, Underwood, Rappolee, & Werb, 1996).

Although nerve graft is considered a standard procedure, yet sacrificing a peripheral nerve is not an ideal option since it can lead to numbness in the receptor zone and may also cause the formation of neuromas and visible scars (Daly, Yao, Zeugolis, Windebank, & Pandit, 2012). Therefore, it is better to find a suitable guidance channel at the site of the nerve gap that can serve as a favorable substrate and nutritional support that cause axonal growth and, consequently, functional recovery. The similarity between the coatings of skeletal muscle fiber with nerve fibers makes it possible to use autologous muscle grafts as a conduit in nerve repair (Roganovic, Ilic, & Savic, 2007).

Clinical recovery and histological evidence of the axonal regeneration has been observed after muscle grafting of the nerve damage in leprosy (de Blaquiere, Curtis, Pereira, & Turk, 1994). Epimysial of degenerated muscle graft as a tube can guide the regenerating nerve fiber to the distal end of the nerve defect (Yang et al., 2013).

To create natural guidance channels that provide favorable conditions for axonal growth and prevent immune responses, it is recommended that a cellular skeletal muscle graft be used in the gap region (Roganovic et al., 2007). Cellular constituents in the skeletal muscle tissue can be removed using various techniques, including initial treatment with alcohol, freezing, and thawing (Roganovic et al., 2007). Exogenous growth factor, along with a nerve conduit, plays a vital role in neuronal survival and nerve regeneration (Ma et al., 2016). It was found that nerve guidance channels containing collagen and laminin-containing gels have better regeneration capacity compared to those that contain only a saline medium (Verdú et al., 2002). Moreover, various isoforms of laminin, as initial compounds of the basal lamina, are considered potent stimulants for axonal growth and repair (Chen & Strickland, 2003). Therefore, in the present research, the effect of a cellular skeletomuscular graft with nerve growth factor and laminin are studied on axonal regeneration and functional recovery following sciatic nerve transection.

## 2. Methods

### 2.1. Animals and groups

This experimental research was done according to the guidelines of Iranian Syndicate for Application and Care of Animals and was approved by the Animal Research Ethics Committee of Yasuj University of Medical Sciences. Forty-two female albino rats of the Wistar strains (Weight: 200±250 g) were purchased from the animal house of the Shiraz University of Medical Sciences. The animals were housed under conditions of controlled temperature (22°C±2°C) with 12-h light/12-h dark cycle and food and water ad libitum. The animals were randomly assigned into three equal groups of 14: the muscle graft+NGF+laminin group, the muscle graft group, and the control group.

### 2.2. Surgical procedure and muscle autografts

The rats were anesthetized via intraperitoneal injection of ketamine (100 mg/kg) and xylazine (10 mg/kg). An incision was made on the posterior surface of the left thigh from the sciatic notch to the popliteal region, the muscles and fascia were pushed away, and the sciatic nerve was exposed. Under aseptic condition at mid-thigh level, 10 mm of the sciatic nerve was cut and removed.

A 12-mm narrow strip of the left gluteus superficialis muscle was removed in alignment with the lengths of the muscle fibers. The narrow strip was placed in liquid nitrogen for 5 minutes to freeze completely, then put in normal saline solution for more than 5 minutes at room temperature. Next, it was placed in sterile distilled water for 10 minutes so that the cytoplasm and cell membrane were removed due to the osmotic phenomenon (through leakage) from the muscle (Glasby, Gschmeissner, Huang, De Souza, 1986). Following that, a surgical blade was used to trim the thawed muscle in the form of a square block (2×2×12 mm) under a stereomicroscope.

One millimeter of both stumps of the distal and proximal sciatic nerve was placed inside muscular graft, and the epimysium of the muscle was sutured to the epineurium of the sciatic nerve at the proximal and distal ends of the nerve with using absorbable suture 10/0 (Ethicon). In the muscle graft+NGF+laminin group, 100 ng of the nerve growth factor (NGF) (Sigma-Aldrich, USA) followed by (1.28 mg/mL) laminin (Sigma) were injected into the muscle graft (Labrador, Butí, & Navarro, 1998). In the muscle graft group, normal saline was injected into the muscle graft. The 10-mm sciatic nerve was transected and removed with the same procedure without any treatment in the control group.

### 2.3. Walking track analysis

Assessment of the functional recovery was done 1, 8, 15, 22, 29, and 60 days after the operation with the measurement of the Sciatic Functional Index (SFI) (Bain, Mackinnon, & Hunter, 1989). The rats’ hind limbs were dipped in Iranian ink and permitted to walk in a wooden box (70×30×20) that covered with a sheet of white paper to record the footprints. The SFI was calculated on the operated and healthy legs of the rats based on the formula introduced by Bain et al. (Bain et al., 1989):

SFI=−38.3(EPL-NPL)/NPL+109.5(ETS-NTS)/NTS+13.3(EIT-NIT)/NIT−8.8

where the Print Length (PL), toe spread from the first to the fifth Toe (Ts), and the Intermediary Toe spread (IT) from the second to the fourth toe were measured on the experimental (EPL, ETS, and EIT) and normal sides (NPL, NTS, and NIT). If SFI is equal to or less than –100, the motor function has been completely lost, values between –100 and –10 indicate an improving trend in the motor function, and those between –10 and +10 represent a normal function of the legs.

### 2.4. Retrograde tracing of spinal motor neurons

Sixty days after treatment, eight rats from each group were anesthetized, and the gastrocnemius muscles on the left side were exposed. 1, 1′-Dioctadecyl-3, 3, 3′, 3 –tetramethylindocarbocyanine Perchlorate (DiI) (Molecular Probes, Leiden, The Netherlands; cat. No, D-282) was used for labeling the spinal motor neurons and evaluation of the axonal regrowth. With using 10-μL Hamilton syringe, 8–9 μL of DiI tracer in 170 mg/mL DMSO was diluted 1:10 in saline and injected into 5 different locations in the bulk of the gastrocnemius muscle (Madison & Robinson, 2014). Ten days after application, the animals were perfused, and their spinal cord segment (L4–L6) was dissected out. Each spinal segment was serially sectioned into 50-μm horizontal sections on a freezing microtome (Leica cryostat). The labeled spinal motor neurons were counted using fluorescent microscopy (Olympus Ax70). As previously described, in each spinal segment, the number of labeled motor neurons of each section summed up together to give the total number of motor neurons for each rat (Catapano et al., 2016).

### 2.5. Histological study

After 60 days post-treatment, 6 rats from each group were anesthetized with double doses of ketamine (200 mg/kg) and xylazine (20 mg/kg) and perfused for histological studies. For counting of myelinated nerve fibers at the grafted region, 12 mm of the sciatic nerve with the center of the transplanted area was cut and removed. This segment was divided into three equal 4-mm parts of proximal, middle, and distal. The proximal parts of each segment were marked to distinguish the distal end from the proximal one. For counting and morphological study of the spinal motor neurons that participated in the sciatic nerve, 5 mm of the L4–L6 spinal cord segment was cut and removed. The spinal cord and each segment of the sciatic nerve were cut into the 50 μm and 5 μm segments, respectively (Leica cryostat, CM 3000).

The number of myelinated nerve fibers and motor neurons in each segment of the spinal cord or sciatic nerve was counted under a microscope (Olympus Bx51). The images of the section were taken with a digital camera (DP 11) connected to the microscope. As previously described (Takami et al., 2002), the number of nerve fibers or neurons of each section was summed up to obtain the final number of nerve fiber or neuron in a 5-mm long spinal cord or 4-mm sciatic nerve segment in each rat.

### 2.6. Statistical analysis

All statistical analyses were performed in SPSS 18.0 (SPSS Inc., USA). One-way ANOVA followed by post hoc Tukey’s were used for data analysis. All data are expressed as Mean±SD. P<0.05 was considered to be statistically significant.

## 3. Results

### 3.1. Gait analysis

**Figure 1.**
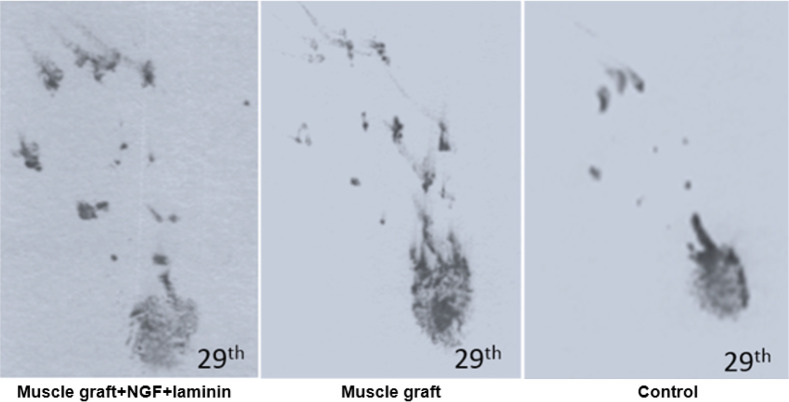
Footprint recorded on the 29^th^ day on the injured left foot

Assessment of the functional recovery was conducted by a researcher who was blind to animal grouping. One day after the operation, the rats dragged their feet with foot drop, and toe adduction in all three groups. Based on Table 1, gait analysis on day 1 and 8 in the different groups indicates that function of the sciatic nerve has been lost, and 1-way ANOVA analyses demonstrate no significant differences between the three groups on the injured foot (P>0.05). The ability to walk on the operated legs in the muscle graft+NGF+laminin and muscle graft groups improved on day 29, and the mean value of SFI increased significantly in these two groups compared to the control group (P<0.05) (Figure 1).

**Table 1.** The Mean±SD values of the Sciatic Functional Index (SFI) in different groups

**Days**	**1**	**8**	**15**	**22**	**29**	**60**

**Group**						
Muscle graft+NGF+laminin	−103.3±3.8	−96.6±2.9	−96.4±3.9	−95.6±3.6	−82.8±3.4 ^*^	−76.6±2.9 ^*^
Muscle graft	−102.4±3.2	−98.8±3.1	−97.9±3.4	−95.8±3.7	−86.01±2.2 ^*^	−82.1±3.5 ^*^
Control	−104.2±2.3	−99.8±2.9	−99.3±2.9	−99.3±2.7	−98.6±3.4	−96.9±2.1

* A significant difference compared with the control group (P<0.01); n=14

Sixty days after the operation, the treated group with muscle graft+NGF+laminin on the left hind limb showed toe-spreading and better footprints walking tracks compared to the other two groups. Statistical analysis on the 60th day indicated that the Mean±SD SFI increased significantly in the muscle graft +NGF+laminin (−76.6±2.9) and muscle graft (−82.1±3.5) groups compared to the control group (−96.9±2.1). Although improvement of functional motor movement in the muscle graft+NGF+laminin group increased compared to the muscle graft group, this difference was not statistically significant. It must be mentioned that one day before the operation, the normal gait was recorded in all three groups and hind limb toes were fully spread, and there were no significant differences between the groups.

### 3.2. Retrograde DiI labeled neuron

**Figure 2.**
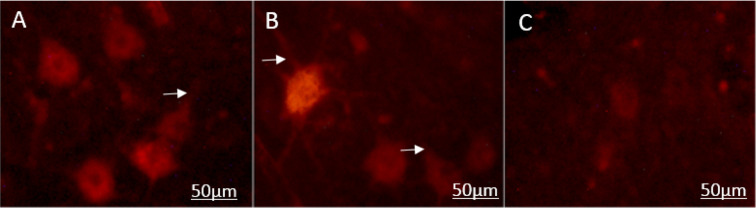
Retrograde DiI labeled motor neurons in different groups A. DiI motor neurons are seen more in the muscle graft+NGF+laminin group; B. Compared to the muscle graft group; C. The control group. The nucleus of the neurons appear darker, and arrow exhibits cytoplasmic process (Magnification×400 in A, B, and C; Scale bar: 50 μm in A, B, and C).

**Figure 3.**
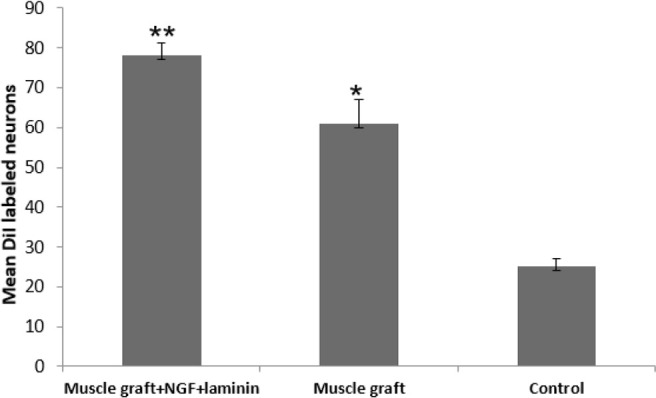
Spinal DiI labeled motorneurons in the spinal cord segment (L4–L6) in each group **P<0.05 compared to the muscle graft group ^**^P<0.001 compared to the control rats; n=8.

DiI positive nerve cells were observed in the perikaryon of the spinal cells through the axonal regrowth 70 days after treatment (Figure 2). DiI tracer in the perikaryon of the motor neuron in the muscle graft+NGF+laminin and muscle graft groups were seen more and more specific than those in the control group (Figure 2). The Mean±SD number of DiI labeled motor neurons in the spinal segment of the L4–L6 in the muscle graft+NGF+laminin, and muscle graft groups were 78.6±3.1 and 61.3±6.1, respectively. One-way ANOVA indicated that there were significant differences compared to the control group (25.2±2.2) (P<0.001) (Figure 3).

### 3.3. Histological studies

**Figure 4.**
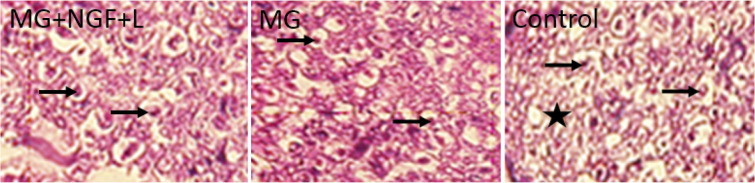
Transverse section of the distal segment in the muscle graft+NGF+laminin rats showed the larger diameter of axons (arrows). Axonal degeneration and myelin break has seen (star) in the control rats.

Histological assessment of the distal segment in the transplanted groups showed that axons could grow and reached the distal part (Figure 4). The thicknesses of myelin of the regenerating axons increased in the muscle graft+NGF+laminin and muscle graft groups compared to that in the control rats (Figure 4).

The Mean±SD number of myelinated nerve fibers in the proximal segment in the muscle graft+NGF+laminin (3353±34.2) and muscle graft (3382±42.6) groups increased compared to that in the control group (3216±53.1) (Table 2). One-way ANOVA analysis showed no difference in the proximal segment between the groups (P>0.05). The Mean±SD number of myelinated nerve fibers in the middle segments in the muscle graft +NGF + laminin (343±11.1) and muscle graft (312±15.9) groups increased significantly compared to that in the control group (200±18.3) (P<0.05). The Mean±SD number of myelinated nerve fibers in the distal segments in the muscle graft+NGF+laminin (18.2±1.6) and muscle graft (14.6±2.1) groups increased significantly compared to that in the control group (6.3±3.4).

**Table 2.** Mean±SD number of myelinated nerve fibers in different groups

**Group**	**No.**	**Proximal**	**Middle**	**Distal**	**Total**
Muscle graft+NGF+laminin	6	3353±34.2	343±11.1^*^	18.2±1.6^**^	4314.2±47.4^*^
Muscle graft	6	3382±42.6	312±15.9^*^	14.6±2.1^**^	3708±39.7
Control	6	3216±53.1	200±18.3	6.3±3.4	3422.3±34.6

* A significant difference compared to control group (P<0.05);

** A significant difference compared to the control group (P<0.01).

**Figure 5.**
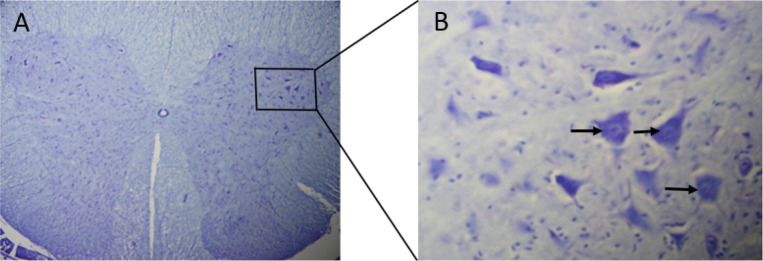
Transverse section of the L4–L6 spinal cord segment A. 60 days post-treatment. High power photomicrographs; B. From the frame show (arrows) that these cells are large, multi-polar with many dendrites and have a coarsely granular cytoplasm (Cresyl violet staining, Scale bars=50 μm in A, 20 μm in B).

Sciatic nerve transection reduced the number of motor neurons in the anterior horn of the injured side compared to that in the healthy side (Figure 5). In each group, the mean number of spinal motor neurons decreased significantly in the left side of the L4–L6 spinal cord segment (The fiber of these neurons were transected in the left sciatic nerve) compared to the right side (P>0.05). The Mean±SD number of the motor neurons in the left side of the spinal segment in the muscle graft+NGF+laminin (475.5±6.1) and muscle graft (403.2±3.8) groups increased significantly compared to that in the control group (219.6±2.2) (Table 3). Although the number of motor neurons in the left side of the spinal segment increased in the muscle graft+NGF+laminin group compared to the muscle graft group, this increase was not significant (P>0.05).

**Table 3.** The Mean±SD number of motor neurons in the right and left side of the anterior horn of the L4–L6 spinal cord segment

**Group**	**No.**	**Motor Neuron**	

		**Right Side**	**Left Side**
Muscle graft+NGF+laminin	6	793.4±3.4 ^*^	475.5±6.1 ^**^
Muscle graft	6	828.5±2.9 ^*^	403.2±3.8 ^**^
Control	6	786.6±3.2 ^*^	219.6±2.2

* A significant difference compared to the Left side motor neuron of the same group (P<0.001);

** A significant difference compared to the control group (P<0.05).

## 4. Discussion

Peripheral nerve injury is a major problem that annually affects millions of people in the world, and most cases require surgical nerve repair (Perretta & Green, 2017). A grafting procedure is necessary to improve the functional recovery when a long segment of the nerve is lost (Mohammadi, Delaviz, Mohammadi, Delaviz, & Rad, 2016). When there is a large gap between the nerve endings, a suitable anatomical conduit is necessary so that the axons in the proximal segment can be guided into the endoneurial tubes of the distal segment and restore the function of the target organ (Iijima, Ajiki, Murayama, & Takeshita, 2016).

To improve and accelerate nerve regrowth, different conduits, including olfactory mucosa, adult Schwann cells, or amniotic membrane with betamethasone were used in experimental studies (Delaviz et al., 2008; Hedayatpour et al., 2007; Sadraie et al., 2016; Zarinfard, Tadjalli, Razavi, & Kazemi, 2016). Among the various attempts, degenerated skeletal muscle autografts could recover the foot and hand sensation in patients with leprosy (Pereira, Bowden, Narayanakumar, & Gschmeissner, 1996). This study confirms the results of other studies that showed the repair of sciatic nerve could enhance the survival of the spinal ganglion cells (Atlasi, Mehdizadeh, Bahadori, & Joghataei, 2009).

Because of the similarity between the tubular matrix of skeletal muscles (the basal membrane) and the endoneurial tubes of a damaged nerve, it is possible to use autologous muscle grafts as a favorable biological conduit for nerve repair (Kang et al., 2015). Therefore, the sheaths of skeletal muscle were employed as a guidance channel in repairing of nerve injury in the clinical and experimental research (Meek, Varejão, & Geuna, 2004; Pereira et al., 1996; Sanes, 2003).

Gait analysis of this study indicated that motor function improved in the groups with degenerated muscle grafts compared to the control group. It has been shown that axonal regeneration could grow from the skeletal muscle grafts and reach the target muscle, and innervate the muscle fibers to improve the motor function (Glasby, Gschmeissner, Huang, & De Souza, 1986). Our study results confirm the Glasby et al. study that demonstrated the use of non-neural autografts and skeletal muscle fibers with parallel array of the nerve fiber could create a matrix of basal membrane tubes that were anatomically and chemically similar to peripheral nerves sheet (Glasby, et al., 1986).

Moreover, Norris et al. showed that the basal membrane tubes derived from skeletal muscles had sufficient diameter to match the largest nerve fibers (Norris, Glasby, Gattuso, & Bowden, 1988). Basal membranes of muscle fibers were in the form of long cylinders lying parallel to each other and provide a suitable substrate for the growing axons (Houštava, Dubový, Haninec, & Grim, 1999). The axons regrowth faster when the conduit matrix of the degenerating muscle is coaxial with the nerve fiber (Glasby, Gschmeissner, Hitchcock, & Huang, 1986; Glasby et al., 1986). Degenerated muscle grafts for nerve repair in primates guide the myelination of the nerve in the graft region and axonal regeneration to the distal nerve segment with the typical electrophysiological results (Glasby, Gschmeissner, & Hitchcock, 1986).

In the present research, functional recovery and axonal regeneration improved in the muscle graft+NGF+laminin group compared to the muscle grafts and the control groups. NGF is essential for the survival of the neuron, synaptic development, axonal regrowth, and functional recovery in the central and peripheral nervous system (Mesentier-Louro et al., 2017). The enriching of the conduit nerve tube with NGF increases the odds of axonal regeneration of a 10-mm long gap of the rat sciatic nerve (Mesentier-Louro et al., 2017). Nerve growth factor by up-regulating p75NTR expression in Schwann cells could stimulate the nerve regrowth in the Wistar rats (Ma, Peng, Wu, Wu, & Gao, 2013). Furthermore, the favorite microenvironment such as laminin and collagen can provide an appropriate matrix for axon regrowth (Cao et al., 2011).

Laminin has biological activities such as neurite-promoting activity and can create a favorable microenvironment for neurotic regeneration in vitro (Manthorpe et al., 1983). Nerve conduction tube filling with substrates such as laminin, fibronectin, and NGF provides a favorable condition for nerve regrowth and cell migration (Glasgow et al., 2016). Suitable concentrations of laminin and growth factors are essential for nerve repair (Valentini, Aebischer, Winn, & Galletti, 1987). The high concentration of laminin inhibits the diffusion of growth factors and act as a barrier for axonal regeneration (Valentini et al., 1987).

Our result confirmed the Labrador et al. study results that showed a low concentration of laminin or collagen could provide better axonal regrowth (Labrador et al., 1998). Laminin-8 and Laminin-2 play an vital role in the development of nerve growth and nerve regeneration after injury (Wallquist et al., 2002). Laminin as a substrate affects Schwann cell migration during nerve repair and has effective role for axonal regeneration in a cellular nerve allograft (Heermann & Schwab, 2013).

Furthermore, laminin with growth factors has a significant role for axonal regeneration in the long gap (20 mm) in the experimental study and NGF increase nerve regrowth and myelin formation (Barbon et al., 2016; Dodla & Bellamkonda, 2008). Our result confirmed Pu et al. study results that showed enriching of the conduit nerve tube with NGF increased the likelihood of axonal regeneration of a 10-mm long gap of the rat sciatic nerve (Meek, 2000). Biological conduit such as combined muscle and vein could reconstruct the digital nerve defect up to 4 cm in patients (Battiston, Geuna, Ferrero, & Tos, 2005). Although many studies do not use an intact group in their research, we think that one limitation of this study was the lack of an intact group that results can be compared with them.

No single drug or particular approach is available for the favorable and sound treatment of peripheral nerve injury. When a long part of nerve continuity is lost, one technique alone cannot provide adequate treatment for nerve repair and combined therapy with conduit tube might achieve better outcomes. Results of this study demonstrated that using biological tubulization such as degenerated skeletal muscle autografts with NGF and laminin could provide a suitable scaffold for bridging a nerve defect. This method is easy to use, less expensive, and without inflammatory responses or complications.
